# Prediction of one-year cognitive decline via self-perceived memory in older adults during the COVID-19 pandemic

**DOI:** 10.1016/j.jarlif.2026.100060

**Published:** 2026-01-24

**Authors:** Kenichiro Sato, Yoshiki Niimi, Ryoko Ihara, Kazushi Suzuki, Atsushi Iwata, Takeshi Iwatsubo

**Affiliations:** a Dementia Inclusion and Therapeutics, The University of Tokyo Hospital, Hongo 7-3-1, Bunkyo-ku, Tokyo, 113-8655, Japan; b Unit for Early and Exploratory Clinical Development, The University of Tokyo Hospital, Hongo 7-3-1, Bunkyo-ku, Tokyo, 113-8655, Japan; c Department of Neurology, Tokyo Metropolitan Institute for Geriatrics and Gerontology, Sakaecho 35-2, Itabashi-ku, Tokyo, 173-0015, Japan; d Division of Neurology, Internal Medicine, National Defense Medical College, Namini 3-2, Tokorozawa-shi, Saitama, 359–8513, Japan; e National Center of Neurology and Psychiatry, Ogawahigashi-cho 4-1-1, Kodaira-shi, Tokyo, , 187-8551, Japan

**Keywords:** Dementia, Self-perception, Cognitive decline, Subjective memory complaints, COVID-19, Cognitive Function Instrument

## Abstract

**Background:**

Subjective memory complaints (SMC) are associated with increased risk of cognitive decline and dementia. SMCs are also influenced by mood and anxiety symptoms, which increased among older adults during the COVID-19 pandemic.

**Objective:**

We investigated whether a single-item SMC measure predicts one-year cognitive decline and whether this predictive relationship remained stable during the early COVID-19 period (2020).

**Methods:**

We analyzed longitudinal data (2011–2023) from the National Health And Aging Trends Study (NHATS), an annual survey of Medicare beneficiaries in the United States. Mixed-effects models assessed the association between self-perceived memory worsening and next-year cognitive outcomes, and tested whether this association differed in 2020.

**Results:**

A total of 33,244 observations from 6676 elderly individuals were included. Higher age, male sex, non-white race, education at or below high school level, lower scores on basic and instrumental activities of daily living, poorer social activity, anxiety symptoms, lower annual income, and self-perceived memory decline each predicted worse cognitive function one year later. However, the interaction between self-perceived memory decline and the COVID-19 pandemic (in 2020) was not significant.

**Discussion:**

A single-item measure of self-perceived memory decline effectively predicts next-year cognitive decline in older adults, independent of the broader social impacts observed during the 2020 COVID-19 pandemic.

## 1 Introduction

Subjective memory complaints (SMC) are known as an important marker of cognitive decline and the development of dementia [1], and in some cohorts are associated with Alzheimer’s disease (AD) biomarker [[2], [3], [4], [5]]. A relevant index is the Cognitive Function Index (CFI) [6], which includes a simple question about self-perceived memory decline “*Compared to one year ago, do you feel that your memory has declined substantially?*”, and it has been demonstrated as a useful tool to predict cognitive decline and incident dementia, and in biomarker-focused cohorts, some items are associated with AD biomarkers [7,8]. Meanwhile, SMC and dementia are also associated with psychiatric symptoms including depression and anxiety [[9], [10], [11]], which means we need to account for these psychiatric symptoms as confounders when considering the association between SMC and the development of dementia.

The outbreak of the new coronavirus (SARS-CoV-2) infection (COVID-19) pandemic since 2020 has brought about a temporary but enormous social and economic impact worldwide [12]: citizens had to change their lifestyles and behaviors, such as social distancing, wearing masks, washing hands, refraining from going out or eating out, working from home, or canceling large-scale events [13,14]. Elderly individuals, who are known to have an increased risk of severe illness or mortality from the COVID-19 infection [15], were also affected [16,17], and many studies reported increased psychological symptoms including depression or anxiety among elderly individuals following pandemic-related changes (e.g., quarantine or decreased physical/social activities) [[18], [19], [20]].

These observations lead us to hypothesize that the interpretation of SMC may be altered to an uncertain extent during the pandemic, mediated by the increased depression or anxiety among elderly individuals as potential confounders in the relationship between SMC and dementia. This can be particularly problematic when analyzing the CFI or other SMC-related assessments in a longitudinal study for AD [6,[21], [22], [23]], which began before the pandemic and is still ongoing. If societal disruption substantially altered the interpretation of SMC, the timing of measurement could matter for longitudinal studies; therefore, we tested whether the association between SMC and next-year cognitive outcomes differed in 2020.

In this study, we aimed to examine this hypothesis using large longitudinal data on thousands of elderly individuals from the National Health And Aging Trends Study (NHATS), which collects annual interviews of nationally representative samples of older adults among Medicare beneficiaries in the United States from 2011 to 2023. The survey collects answers to a question about the self-perceived memory decline (“*Compared to one year ago, would you say your memory is much better now, better now, about the same, worse now, or much worse now than it was then?*”), similar to the one in the CFI. Thus, we expect we can measure the effect of this self-perception question on predicting cognitive decline in the near future, in the context of the NHATS study. We therefore asked whether this single-item SMC measure predicts one-year cognitive decline and whether its predictive validity changed in 2020. Because NHATS does not include AD biomarkers or adjudicated etiologic dementia diagnoses, our outcomes reflect cognitive test–based measures (including NHATS-defined possible/probable dementia classifications) rather than confirmed AD pathology.

## 2 Methods

### 2.1 Study design

This is a retrospective study using publicly available data from the NHATS study (https://www.nhats.org). The NHATS study has conducted nationwide surveys conducted since 2011, comprising annual interviews with over 10,000 Medicare beneficiaries aged ≥ 65 years in the United States, to investigate late-life disability trends and trajectories (https://www.nhats.org) [24]. Stratified random sampling of community-dwelling older adults has been applied to select participants: the detailed study design and procedures are described in their previous technical reports by the study team [25]. The data have been distributed annually, and we obtained the data (Rounds 1–13) from their website (https://nhats.org/researcher/data-access/public-use-files) in 2025.

### 2.2 Cognitive function in NHATS

In NHATS, three cognitive function domains have been collected each round from participants: an orientation assessment consisting of eight questionnaires (i.e., today’s day of week, day, month, year, and the name of current president and vice president of the United States) with scores ranging from 0 to 8 (higher score for better function); a 10-word recall test to evaluate memory recall (score range 0–20, with higher scores indicating better function); and clock-drawing test (CDT) to assess executive function (score range 0–5, with higher scores indicating better function) [25]. The cut-off threshold for significant decline in the study population is reported as the mean minus 1.5 SD [25]–namely, scores of 0–3 in orientation, 0–3 in recall, and 0–1 in CDT. These cognitive tests were administered to all participants every round. According to the study team report [25], participants whose cognitive scores significantly decline in any one domain are considered to have possible dementia, while those with declines in two or three domains are considered to have probable dementia. There are no biomarkers or adjudicated etiologic dementia diagnoses available in NHATS for this analysis to determine underlying AD pathology.

### 2.3 Statistical analyses

Data handling and analyses were conducted using R statistical software (version 4.1.0, The R Foundation for Statistical Computing). We employed a mixed-effects model to conduct one-year predictions–that is, using explanatory variables measured in the current year to predict cognitive status in the following year, independent of other years (e.g., using Round 1 variables to predict cognitive decline in Round 2). Thus, we referred to the Round 1–12 data, aggregating them into a single dataset [11] to apply a mixed-effects model with repeated measures.

For each round, the following three types of next-year target variables were used in mixed-effects regression based on the current-year explanatory variables:

1) Binary outcome indicating the presence or absence of possible dementia in the following round (logistic regression)
2) Binary outcome indicating the presence or absence of probable dementia in the following round (logistic regression)
3) Count of cognitive domains (0–3) showing significant decline in the following round (zero-inflated Poisson regression [26])

The model formula (1) is as follows (details of the other model formulae are provided in Table S1): $\log\left(Odds\;of\;possible\;dementia\;in\;the\;next\;year\right)=\beta_{0}+\beta_{1}Age+\beta_{2}Sex+\beta_{3}White+\beta_{4}Black+\beta_{5}Household+\beta_{6}Education+\beta_{7}Care+\beta_{8}bADL+\beta_{9}iADL+\beta_{10}PHQ2+\beta_{11}GAD2+\beta_{12}PA+\beta_{13}SA+\beta_{14}Income+\beta_{15∼21}MHX_{1∼7}+\beta_{22}PossibleDementia+\beta_{23}Round+\beta_{24}SMC+\beta_{25}\left(SMC\;\times Year2020\right)+\gamma_{1}+\gamma_{2}+\gamma_{3}$.

In the above equation, β_0_ is the fixed intercept, β_1_ through β_25_ are fixed effects, and γ_1_ through γ_3_ represent random intercepts corresponding to participant ID, census division region ID, and a binary variable indicating the presence or absence of comorbidity of AD or dementia on the current year. Fixed effects β_1_ through β_24_ are defined as follows: “*Age*”: 5-year age category (e.g., 50–54, 55–59, etc.); “*Sex*”: male or female; race variables: “*White*” (indicating white, or not) and “*Black*” (indicating black, or not); “*Household*”: living alone or not; “*Education*”: highest degree converted to education years (e.g., 12 years for high school graduation); “*Care*”: residential care status (e.g., community-dwelling, receiving residential care, residing in a nursing home, etc.); “*bADL*”: basic ADL total score; “*iADL*”: instrumental ADL total score; “*PHQ2*”: binary score indicating whether depression score in PHQ-2 ≥ 3 as a screen for depression [27]; “*GAD2*”: binary score indicating whether anxiety score in GAD-2 ≥ 3 as a screen for anxiety [28]; “*PA*”: physical activity score [29]; “*SA*”: social activity score [29]; “*Income*”: reported income category (i.e., < $15,000, $15,000–30,000, ≥ $30,001); “*MHX*s”: indicators for the presence or absence of comorbidities (heart attack, heart diseases, high blood pressure, diabetes, stroke, lung diseases, and cancer, respectively); “*PossibleDementia*”: indicator for possible dementia in the current year; “*ProbableDementia*”: indicator for probable dementia in the current year; “*Domains*”: number of cognitive domains with significant decline in the current year; “*Round*”: current survey round (e.g., Round 1, Round 2, etc.); “*SMC*”: indicates a response of “*Worse*” or “*Much worse*” to the self-perceived memory decline question ”*Compared to one year ago, would you say your memory is much better now, better now, about the same, worse now, or much worse now than it was then?*” (response options: “Much better”, “Better”, “Same”, “Worse”, and “Much Worse”); and the interaction term between “*SMC*“ and “*Year2020*”, a binary flag for the pandemic year (e.g., 1 = 2020 and 0 = other year). This was conducted in a whole case dataset (“Scenario 1”).

We also performed a similar analysis on another dataset in which the current cognitive domains were limited to 0 (“Scenario 2″), while excluding the explanatory variable related to current cognitive status (e.g., excluding the “*PossibleDementia*” term from the formula above). If the 95 % confidence interval (CI) for the estimated coefficient of *SMC* (β_24_) does not include 0, then self-perceived memory decline in a given year would contribute to the prediction of cognitive deterioration in the following year. Similarly, if the lower bound of the 95 % CI for the interaction term (β_25_) is above 0, self-perceived memory during the pandemic year 2020 is additionally associated with cognitive deterioration in the following year (in 2021).

### 2.4 Ethics

This study was approved by the University of Tokyo Graduate School of Medicine institutional ethics committee (ID: 2025264NI). Informed consent was not required because the study uses publicly available, anonymized data only.

## 3 Results

### 3.1 Demographics

The obtained data from Rounds 1 through 13 included 47,415 observations from 8446 unique participants in total. Among them, only those observations with no missing data in the necessary variables for the mixed-effects models were used for the analysis: for Scenario 1 (including all eligible cases), 33,244 observations from 6676 unique participants were included, and for Scenario 2 (including those whose current cognitive domains are all intact), 29,678 observations from 6187 unique participants were included.

Table 1 shows the basic demographics in Scenario 1: the majority of them are White (70 %), 20 % are Black, nearly all receive community-based residential care, approximately one-third live alone, approximately 60 % have 12 or fewer years of education, and they are largely independent in their basic and instrumental ADL. Their frequency of possible and probable dementia in Round 1 was 13.8 % and 2.8 %, respectively.

**Table 1:** Demographics of the Included Unique Participants (*n* = 6676).

Features		Summary
Sex: Female		3669 (55.0 %)
Race: White		4643 (69.5 %)
Race: Black		1392 (20.9 %)
Education Years: ≤ 12 years		3890 (58.7 %)
Round 1^†^	Age Category	65–69 y/o: 22.3 % 70–74 y/o: 23.4 % 75–79 y/o: 21.0 % 80–84 y/o: 19.0 % 85- y/o: 9.8 %
	Living Alone	32.80 %
	Community-Dwelling	96.10 %
	Basic ADL score	Median 0 (IQR: 0∼1)
	Instrumental ADL score	Median 0 (IQR: 0∼1)
	PHQ-2 ≥ 3 (yes)	13.10 %
	GAD-2 ≥ 3 (yes)	11.20 %
	SMC (Answered “Worse” or “Much Worse” to a question about self-perceived memory decline*)	13.20 %
	Physical Activity score	2: 30.5 % 1: 40.1 % 0: 29.4 %
	Social Activity score	0: 3.3 % 1: 10.1 % 2: 23.6 % 3: 28.8 % 4: 20.5 % 5: 13.7 %
	Income Class	<$15,000: 29.3 % $15,000-$30,000: 27.8 % ≥$30,001: 42. %
	Comorbidity: Heart attack	14.50 %
	Comorbidity: Heart disease	17.40 %
	Comorbidity: High blood pressure	67.40 %
	Comorbidity: Diabetes	25.10 %
	Comorbidity: Stroke	9.90 %
	Comorbidity: Lung disease	15.00 %
	Comorbidity: Cancer	25.60 %
	Comorbidity: Dementia	1.90 %
	Orientation score [0–8]	Median 6 (IQR: 5–8)
	Recall score [0–20]	Median 8 (IQR: 6–10)
	Clock Drawing Test score [0–5]	Median 4 (IQR: 3–5)
	Possible Dementia (decreased domains ≥1)	13.80 %
	Probable Dementia (decreased domains ≥2)	2.80 %

Most features denoted by a dagger [†] are those of participants in Round 1, for whom the number of participants does not equal to the total number of participants included in this study. Therefore, to summarize frequencies, we displayed only percentages. A question (“*Compared to one year ago, would you say your memory is much better now, better now, about the same, worse now, or much worse now than it was then?*”) was used as a surrogate marker of SMC (an asterisk [*] in the table).

Abbreviations: SMC, Subjective Memory Complaints; ADL, Activities of Daily Living; PHQ, Patient Health Questionnaire; GAD, Generalized Anxiety Disorder; IQR, Interquartile Range.

### 3.2 Mixed-Effects model results

**Fig. 1:**
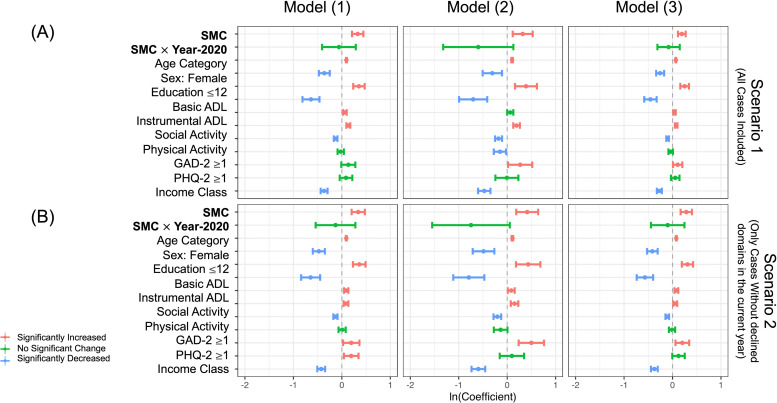
Forest Plot of the Results. Log-transformed coefficients with 95 % CIs are shown. Variables with a significant increase are colored in red, those with a significant decrease in blue, and non-significant variables in green. Abbreviations: SMC, Subjective Memory Complaints; ADL, Activities of Daily Living; PHQ, Patient Health Questionnaire; GAD, Generalized Anxiety Disorder; CI, confidence interval.

Log-transformed coefficients with 95 % CIs are summarized in forest plots (Fig. 1), organized by model and the examined data scenarios, and display some selected variables (full results are shown in Table S2, S3). Variables with a significant increase are colored in red, those with a significant decrease in blue, and non-significant variables in green. Briefly, for all models and both scenarios, the SMC question in a certain year was slightly but significantly associated with the cognitive decline in the next year (e.g., ORs of Models (1)-(3) were approximately 1.2 to 1.5 for those who answered as “Worse” or “Much Worse” to the question [Fig. 1]), while the interaction term between SMC and the pandemic year 2020 was not statistically associated with.

Additionally, higher age (e.g., OR of approximately 1.1 per 1-year increase in age), male sex (e.g., OR of approximately 0.6–0.7 for those who are female), not being White (e.g., OR of approximately 0.5–0.6 for those who are White), having a high school diploma or lower (e.g., OR of approximately 1.3–1.5), lower instrumental ADL (e.g., OR of approximately 1.1 per 1-point worsening in ADL score), poorer social activity (e.g., OR of approximately 1.1–1.2 per 1-point worsening in Social Activity score), having anxiety symptoms (e.g., OR of approximately 1.1 if GAD-2 score ≥ 3), and lower annual income were consistently significantly associated with the worse cognitive function in the subsequent year (Table S2, S3).

## 4 Discussion

Our analysis of NHATS data demonstrates that a single self‐report item assessing memory change–“*Compared to one year ago, would you say your memory is much better now, better now, about the same, worse now, or much worse now than it was then?*”–robustly predicts one‐year cognitive deterioration. This finding supports the notion that simple subjective assessments can serve as one of the early markers of cognitive decline, even when measured against significant societal disruptions such as the COVID-19 pandemic. Notably, the absence of a modifying effect of the pandemic on this relationship, which is compatible with some earlier literature [30], reinforces the resilience of the measure under conditions of widespread pandemic stress.

The similarity between the examined question (“*Compared to one year ago, would you say your memory is much better now, better now, about the same, worse now, or much worse now than it was then?*”) in the NHATS study [24] with the corresponding item (“*Compared to one year ago, do you feel that your memory has declined substantially?*”) in the CFI [6] suggests the utility and significance of the examined question. The longitudinal worsening in CFI score has been shown to be associated with objective cognitive decline [6,21], and the corresponding CFI item has been shown to predict amyloid-PET positivity among cognitively unimpaired individuals [7] (e.g., OR ∼ 1.8 [22]). Consistently, we observed a similar effect size (e.g., OR ∼1.5) for the current question in predicting one-year cognitive decline. These current results can also be interpreted as demonstrating the utility of the CFI index in the NHATS study, which represents a more heterogeneous population than typical clinical trials, and therefore may be more reflective of real-world settings. The simplicity and ease of the question about the self-perceived cognitive decline highlight its potential for wide applicability as a screening tool in real-world practice, beyond clinical trial settings. In practice, rather than relying on this single question alone, integrating the question assessment into routine clinical screenings could facilitate much better prediction of short-term cognitive decline, thereby contributing to earlier interventions and personalized management plans.

The association between short-term cognitive decline (one-year later) and various examined covariates (e.g., increased age, lower education, reduced functional and social activities, and worse anxiety symptoms) is consistent with earlier literature [11,29]. Moreover, the absence of a significant modifying effect of the COVID-19 pandemic implies that this metric retains its predictive power even during periods of widespread societal stress. There were concerns that pandemic-related disruptions could confound the short-term prediction of cognitive decline by self-perceived cognitive worsening, especially when analyzing longitudinal data covering the COVID-19 pandemic period. However, the findings of the present study suggest that this concern may be unwarranted.

This investigation benefits from several notable strengths. The study employs the NHATS database, a large, nationally representative cohort, which enhances the generalizability of the findings. Additionally, the use of repeated annual measurements and robust mixed-effects models allows for controlling intra-individual variability over time. Furthermore, the inclusion of a wide array of covariates—including sociodemographic factors, functional status, and mental health measures—strengthens the validity of the observed associations. These methodological aspects likely contribute to the reliability and clinical relevance of our current study.

Nevertheless, this study has several limitations. First, the self-reported nature of the memory decline measure inherently carries the risk of recall bias and subjective interpretation, which may vary among individuals. Second, the NHATS study primarily involves Medicare beneficiaries, most of whom are White, potentially limiting the generalizability of the findings to non-US populations, especially in Asian regions. Third, NHATS does not include confirmed SARS-CoV-2 infection status for this analysis; therefore, we cannot distinguish post-infectious cognitive sequelae from broader societal stressors (e.g., isolation, anxiety, reduced activity). Accordingly, our results specifically address whether the SMC item’s predictive validity changed in 2020. Fourth, although the analysis used in this study is in a sense an extension of earlier literature [11], additional approaches from the perspective of causal inference may be needed to validate our results. Finally, NHATS does not provide adjudicated dementia diagnoses or AD biomarkers; therefore, these findings pertain to cognitive test–based decline and NHATS-defined possible/probable dementia status rather than confirmed AD pathology.

To further clarify the meaning of this single-item SMC signal, future work should (1) link comparable SMC items to biomarker cohorts (amyloid/tau and/or plasma markers) to test whether pandemic-era distress alters SMC–biomarker coupling; (2) evaluate heterogeneity through subgroup analyses by baseline cognitive status, depression/anxiety strata, and markers of social isolation or activity disruption; and (3) examine longer-horizon outcomes (e.g., 2–3-year cognitive decline) to confirm whether the stability observed for 2020→2021 persists beyond this time window.

In conclusion, this study provides evidence that a single, self-reported question regarding memory decline can serve as a simple predictor of cognitive deterioration one year later. Despite the disruptive context of the COVID-19 pandemic, the predictive capacity of this measure remains robust, offering a practical tool for early identification and timely intervention among older adults at risk for cognitive impairment.

## Consent statement

Informed consent was not necessary for this type of study.

## Funding

This study was supported by JSPS KAKENHI Grant Number JP24K10653 (RI) and JP25K19014 (KS), AMED Grant Number JP24dk0207054 (YN) and 25dk0207075 (KS).

Data Availability

The NHATS data supporting the findings of this study is openly available at (https://www.nhats.org/researcher/nhats).

Declaration of the use of generative AI and AI-assisted technologies in scientific writing and in figures, images and artwork

The authors used cloud large language models (ChatGPT and Gemini) for English proofreading. After using these tools, the authors reviewed and edited the content as needed and take full responsibility for the content of the published article.

## CRediT authorship contribution statement

**Kenichiro Sato:** Writing – original draft, Methodology, Investigation, Formal analysis, Data curation, Conceptualization. **Yoshiki Niimi:** Writing – review & editing, Conceptualization. **Ryoko Ihara:** Writing – review & editing, Conceptualization. **Kazushi Suzuki:** Writing – review & editing. **Atsushi Iwata:** Writing – review & editing. **Takeshi Iwatsubo:** Writing – review & editing, Supervision.

## Declaration of competing interests

The authors declare the following financial interests/personal relationships which may be considered as potential competing interests:

Takeshi Iwatsubo reports financial support was provided by Effissimo Capital Management Pte Ltd. Takeshi Iwatsubo reports financial support was provided by Japan Agency for Medical Research and Development. Kenichiro Sato reports financial support was provided by Japan Society for the Promotion of Science. If there are other authors, they declare that they have no known competing financial interests or personal relationships that could have appeared to influence the work reported in this paper.

## Appendix Supplementary materials

Image, application 1
