# Supplementary material for: Prediction of one-year cognitive decline via self-perceived memory in older adults during the COVID-19 pandemic
Source: JAR Life. 2026 Jan 24;15:100060. doi: 10.1016/j.jarlif.2026.100060 (PMC12860612; doi:10.1016/j.jarlif.2026.100060)
Supplement: Supplementary file 1 [file mmc1.docx]

Supplemental Materials

**Table S1. Model Formulae**

| Model # | Link Function | Formula |
| --- | --- | --- |
| (1) | Logistic | $\ln\left( \boldsymbol{Odds of possible dementia in the next year} \right)=\beta_{0}+\beta_{1}Age+\beta_{2}Sex+\beta_{3}White+\beta_{4}Black+\beta_{5}Household+\beta_{6}Education+\beta_{7}Care+\beta_{8}bADL+\beta_{9}iADL+\beta_{10}PFQ2+\beta_{11}GAD2+\beta_{12}PA+\beta_{13}SA+\beta_{14}Income+\beta_{15\sim21}{MHX}_{1\sim7}+\beta_{22}\boldsymbol{PossibleDementia}+\beta_{23}Round+\beta_{24}SMC+\beta_{25}(SMC \times Year2020)+\gamma_{1}+\gamma_{2}+\gamma_{3}$. |
| (2) | Logistic | $\ln\left( \boldsymbol{Odds of probable dementia in the next year} \right)=\beta_{0}+\beta_{1}Age+\beta_{2}Sex+\beta_{3}White+\beta_{4}Black+\beta_{5}Household+\beta_{6}Education+\beta_{7}Care+\beta_{8}bADL+\beta_{9}iADL+\beta_{10}PFQ2+\beta_{11}GAD2+\beta_{12}PA+\beta_{13}SA+\beta_{14}Income+\beta_{15\sim21}{MHX}_{1\sim7}+\beta_{22}\boldsymbol{ProbableDementia}+\beta_{23}Round+\beta_{24}SMC+\beta_{25}(SMC \times Year2020)+\gamma_{1}+\gamma_{2}+\gamma_{3}$. |
| (3) | Zero-Inflated Poisson | $\mathbf{The number of domains with singificant decline}=\beta_{0}+\beta_{1}Age+\beta_{2}Sex+\beta_{3}White+\beta_{4}Black+\beta_{5}Household+\beta_{6}Education+\beta_{7}Care+\beta_{8}bADL+\beta_{9}iADL+\beta_{10}PFQ2+\beta_{11}GAD2+\beta_{12}PA+\beta_{13}SA+\beta_{14}Income+\beta_{15\sim21}{MHX}_{1\sim7}+\beta_{22}\boldsymbol{Domains}+\beta_{23}Round+\beta_{24}SMC+\beta_{25}(SMC \times Year2020)+\gamma_{1}+\gamma_{2}+\gamma_{3}$. |

The differences among the three formulae are highlighted in bold.

Abbreviations: SMC, Subjective Memory Complaints; ADL, Activities of Daily Living; PHQ, Patient Health Questionnaire; GAD, Generalized Anxiety Disorder; PA, Physical Activity; SA, Social Activity; MHX, Comorbidities.

**Table S2. Analysis Results for Scenario 1**

|  | Model (1) | | | | Model (2) | | | | Model (3) | | | |
| --- | --- | --- | --- | --- | --- | --- | --- | --- | --- | --- | --- | --- |
|  | OR | Lower 95% | Upper 95% |  | OR | Lower 95% | Upper 95% |  | IRR | Lower 95% | Upper 95% |  |
| (Intercept) | 0.001 | 0.000 | 0.003 | * | 0.000 | 0.000 | 0.001 | * | 0.002 | 0.001 | 0.005 | * |
| Age Category | 1.098 | 1.090 | 1.107 | * | 1.107 | 1.091 | 1.122 | * | 1.071 | 1.064 | 1.077 | * |
| Sex: Female | 0.696 | 0.623 | 0.779 | * | 0.736 | 0.602 | 0.899 | * | 0.775 | 0.714 | 0.841 | * |
| Race: White | 0.529 | 0.444 | 0.630 | * | 0.496 | 0.371 | 0.663 | * | 0.634 | 0.558 | 0.721 | * |
| Race: Black | 0.960 | 0.796 | 1.158 |  | 0.953 | 0.705 | 1.289 |  | 0.970 | 0.849 | 1.108 |  |
| Living Alone | 0.853 | 0.766 | 0.950 | * | 0.831 | 0.682 | 1.013 |  | 0.930 | 0.861 | 1.005 |  |
| Education years ≤ 12 | 1.422 | 1.263 | 1.601 | * | 1.475 | 1.176 | 1.848 | * | 1.284 | 1.174 | 1.404 | * |
| Residential: Community | 0.537 | 0.440 | 0.656 | * | 0.433 | 0.315 | 0.594 | * | 0.661 | 0.578 | 0.757 | * |
| Basic ADL | 1.065 | 1.025 | 1.106 | * | 1.065 | 1.000 | 1.135 |  | 1.042 | 1.017 | 1.068 | * |
| Instrumental ADL | 1.143 | 1.098 | 1.191 | * | 1.214 | 1.133 | 1.300 | * | 1.078 | 1.050 | 1.107 | * |
| PHQ-2 ≥ 3 | 1.089 | 0.956 | 1.241 |  | 0.994 | 0.784 | 1.260 |  | 1.057 | 0.968 | 1.155 |  |
| GAD-2 ≥ 3 | 1.142 | 0.989 | 1.320 |  | 1.307 | 1.019 | 1.676 | * | 1.112 | 1.010 | 1.225 | * |
| SMC | 1.386 | 1.233 | 1.559 | * | 1.378 | 1.121 | 1.694 | * | 1.212 | 1.119 | 1.312 | * |
| Physical Activity | 0.977 | 0.915 | 1.043 |  | 0.861 | 0.759 | 0.977 | * | 0.963 | 0.919 | 1.009 |  |
| Social Activity | 0.880 | 0.847 | 0.914 | * | 0.837 | 0.779 | 0.899 | * | 0.903 | 0.879 | 0.928 | * |
| Income Class | 0.693 | 0.646 | 0.744 | * | 0.623 | 0.548 | 0.708 | * | 0.762 | 0.723 | 0.802 | * |
| Comorbidity: Heart Attack | 1.232 | 1.017 | 1.492 | * | 1.060 | 0.734 | 1.529 |  | 1.074 | 0.940 | 1.228 |  |
| Comorbidity: Heart Disease | 0.885 | 0.784 | 0.999 | * | 0.682 | 0.543 | 0.857 | * | 0.909 | 0.832 | 0.992 | * |
| Comorbidity: High Blood Pressure | 0.880 | 0.781 | 0.992 | * | 0.696 | 0.561 | 0.862 | * | 0.897 | 0.822 | 0.979 | * |
| Comorbidity: Diabetes | 1.038 | 0.925 | 1.164 |  | 0.822 | 0.667 | 1.015 |  | 1.048 | 0.963 | 1.140 |  |
| Comorbidity: Stroke | 1.030 | 0.831 | 1.276 |  | 0.969 | 0.663 | 1.417 |  | 1.027 | 0.892 | 1.183 |  |
| Comorbidity: Lung Disease | 0.856 | 0.754 | 0.972 | * | 0.769 | 0.610 | 0.968 | * | 0.907 | 0.827 | 0.995 | * |
| Comorbidity: Cancer | 0.965 | 0.826 | 1.128 |  | 0.881 | 0.642 | 1.209 |  | 0.986 | 0.881 | 1.103 |  |
| Cognitive Status in the current year (†) | 4.259 | 3.862 | 4.697 | * | 16.849 | 13.501 | 21.028 | * | 1.874 | 1.778 | 1.975 | * |
| Round identifier | 0.973 | 0.959 | 0.987 | * | 0.977 | 0.951 | 1.005 |  | 0.975 | 0.965 | 0.986 | * |
| SMC $\times$ Year 2020 | 0.942 | 0.665 | 1.333 |  | 0.551 | 0.267 | 1.136 |  | 0.922 | 0.732 | 1.161 |  |

For cognitive status in the current year (dagger [†]), *PossibleDementia* for model (1), *ProbableDementia* for model (2), and *Domains* for model (3) were imputed. Asterisks (*) mean significant change of the variables.

Abbreviations: OR, odds ratio; IRR, incidence rate ratio; SMC, Subjective Memory Complaints; ADL, Activities of Daily Living; PHQ, Patient Health Questionnaire; GAD, Generalized Anxiety Disorder.

**Table S3. Analysis Results for Scenario 2**

|  | Model (1) | | | | Model (2) | | | | Model (3) | | | | |
| --- | --- | --- | --- | --- | --- | --- | --- | --- | --- | --- | --- | --- | --- |
|  | OR | Lower 95% | Upper 95% |  | OR | Lower 95% | Upper 95% |  | IRR | Lower 95% | Upper 95% |  |  |
| (Intercept) | 0.001 | 0.000 | 0.002 | * | 0.000 | 0.000 | 0.001 | * | 0.001 | 0.000 | 0.002 | * |  |
| Age Category | 1.096 | 1.087 | 1.106 | * | 1.114 | 1.097 | 1.132 | * | 1.082 | 1.073 | 1.090 | * |  |
| Sex: Female | 0.622 | 0.549 | 0.705 | * | 0.614 | 0.491 | 0.768 | * | 0.660 | 0.591 | 0.736 | * |  |
| Race: White | 0.525 | 0.432 | 0.638 | * | 0.453 | 0.328 | 0.624 | * | 0.566 | 0.478 | 0.670 | * |  |
| Race: Black | 0.900 | 0.729 | 1.113 |  | 1.010 | 0.726 | 1.406 |  | 0.891 | 0.743 | 1.067 |  |  |
| Living Alone | 0.854 | 0.756 | 0.964 | * | 0.820 | 0.660 | 1.019 |  | 0.892 | 0.802 | 0.992 | * |  |
| Education years ≤ 12 | 1.432 | 1.257 | 1.632 | * | 1.542 | 1.199 | 1.984 | * | 1.361 | 1.212 | 1.528 | * |  |
| Residential: Community | 0.619 | 0.489 | 0.782 | * | 0.439 | 0.307 | 0.628 | * | 0.682 | 0.558 | 0.834 | * |  |
| Basic ADL | 1.094 | 1.046 | 1.145 | * | 1.087 | 1.012 | 1.167 | * | 1.084 | 1.043 | 1.126 | * |  |
| Instrumental ADL | 1.090 | 1.038 | 1.144 | * | 1.164 | 1.077 | 1.258 | * | 1.057 | 1.013 | 1.102 | * |  |
| PHQ-2 ≥ 3 | 1.213 | 1.042 | 1.411 | * | 1.102 | 0.857 | 1.416 |  | 1.132 | 0.995 | 1.287 |  |  |
| GAD-2 ≥ 3 | 1.214 | 1.025 | 1.437 | * | 1.646 | 1.268 | 2.138 | * | 1.224 | 1.063 | 1.410 | * |  |
| SMC | 1.403 | 1.223 | 1.608 | * | 1.512 | 1.206 | 1.895 | * | 1.329 | 1.182 | 1.494 | * |  |
| Physical Activity | 1.005 | 0.933 | 1.083 |  | 0.874 | 0.759 | 1.006 |  | 0.990 | 0.928 | 1.056 |  |  |
| Social Activity | 0.876 | 0.839 | 0.914 | * | 0.816 | 0.754 | 0.883 | * | 0.898 | 0.865 | 0.932 | * |  |
| Income Class | 0.653 | 0.602 | 0.707 | * | 0.552 | 0.479 | 0.636 | * | 0.688 | 0.641 | 0.738 | * |  |
| Comorbidity: Heart Attack | 1.305 | 1.044 | 1.630 | * | 1.044 | 0.699 | 1.560 |  | 1.213 | 1.005 | 1.464 | * |  |
| Comorbidity: Heart Disease | 0.861 | 0.751 | 0.986 | * | 0.726 | 0.566 | 0.932 | * | 0.862 | 0.765 | 0.971 | * |  |
| Comorbidity: High Blood Pressure | 0.944 | 0.825 | 1.079 |  | 0.681 | 0.537 | 0.865 | * | 0.948 | 0.842 | 1.066 |  |  |
| Comorbidity: Diabetes | 1.159 | 1.021 | 1.315 | * | 0.814 | 0.645 | 1.026 |  | 1.130 | 1.011 | 1.262 | * |  |
| Comorbidity: Stroke | 1.114 | 0.864 | 1.437 |  | 1.080 | 0.713 | 1.637 |  | 1.084 | 0.875 | 1.342 |  |  |
| Comorbidity: Lung Disease | 0.907 | 0.787 | 1.045 |  | 0.807 | 0.627 | 1.040 |  | 0.940 | 0.831 | 1.063 |  |  |
| Comorbidity: Cancer | 0.965 | 0.807 | 1.154 |  | 0.829 | 0.584 | 1.176 |  | 0.968 | 0.829 | 1.131 |  |  |
| Round identifier | 0.976 | 0.960 | 0.992 | * | 0.975 | 0.946 | 1.006 |  | 0.980 | 0.966 | 0.995 | * |  |
| SMC $\times$ Year 2020 | 0.878 | 0.583 | 1.322 |  | 0.474 | 0.213 | 1.057 |  | 0.905 | 0.640 | 1.280 |  |  |

Asterisks (*) mean significant change of the variables.

Abbreviations: OR, odds ratio; IRR, incidence rate ratio; SMC, Subjective Memory Complaints; ADL, Activities of Daily Living; PHQ, Patient Health Questionnaire; GAD, Generalized Anxiety Disorder.
